# Rethinking Sexual Trauma Research: University Students Reactions to Participating in a Sexual Trauma Survey

**DOI:** 10.1177/08862605251319293

**Published:** 2025-02-18

**Authors:** Megan Reynolds, Ngozi Anyadike-Danes, Susan Lagdon, Áine Aventin, William F. Flack, Emily McGlinchey, Chérie Armour

**Affiliations:** 1 Anti-Bullying Centre, Dublin City University, Dublin, Ireland; 2 Ulster University, Belfast, Northern Ireland; 3 Ulster University, Coleraine, Northern Ireland; 4 Queens University Belfast, Belfast, Northern Ireland; 5 Bucknell University, Lewisburg, PA, USA

**Keywords:** ethics, sexual violence, sexual trauma, reactions to research participation, university students

## Abstract

The consistently high prevalence of unwanted sexual experiences (USEs) on university campuses has led to increasing calls for evidence-based solutions to inform policies, training, and intervention development. However, Research Ethics Committees are often hesitant to approve sexual trauma research due to beliefs that asking participants about traumatic experiences will cause extreme distress. Conversely, previous literature has found that many participants who have experienced sexual trauma report positive reactions following their participation in such research. Studies have found that while immediate negative emotional reactions are common, this distress is short-term (e.g., lasting only minutes or hours after participation). The present study assessed 469 Northern Irish university students’ experiences of participating in research addressing USEs. The findings indicated that participating in USE research was a positive experience for participants, regardless of victimization status. Further, participants who reported a victimization experience did not report experiencing a negative emotional reaction to participating in the study. This article considers the ethics of conducting sexual trauma research among university students, with reference to common ethical concerns that can be addressed as part of the research process.

## Introduction

Given the wealth of sexual trauma research among university students, particularly with the emerging research from the United Kingdom and Ireland, it is timely to revisit the role of ethics in this research (Downes et al., 2014). Research Ethics Committees (REC) [U.K. and Ireland], or Institutional Review Boards as known in the United States of America, have an important purpose to safeguard both participants and researchers, enhancing ethical and scientific quality, abiding by their country’s laws and regulations, as well as offering formal accountability at both the institutional and practitioner/researcher level (Page & Nyeboer, 2017; Shaw et al., 2005). Given that a major role of RECs is to identify potential risks to participants and determine whether the benefits from the study outweigh any potential harms (Kuyper et al., 2012), it is perhaps unsurprising that some RECs raise concerns and are often hesitant in approving sexual-trauma-based research (Clark & Walker, 2011; Edwards et al., 2017; Yeater et al., 2012). Therein lies a conundrum: if RECs deny approval to sexual trauma studies, knowledge and understanding regarding sexual trauma and related issues will be limited, which in turn stifles evidence-based practice and support for those most in need (Becker-Blease & Freyd, 2006; Jaffe et al., 2015).

Researchers have reported and discussed how some RECs routinely argue that research with survivors of sexual trauma might be too sensitive and participants too vulnerable to participate (Becker-Blease & Freyd, 2006; Coleman, 2009; Yeater et al., 2012), resulting in unwarranted distress (e.g., questions are too personal, experienced negative emotions, etc.) and possible re-traumatization (Shorey et al., 2011). However, there are two rebuttals to this position: (a) there is insufficient evidence regarding participants’ experiences and reactions to sexual trauma research to support this position, and (b) a lack of clarity and understanding of the risks and benefits of such research for RECs and researchers to take this position (Carter-Visscher et al., 2007).

The literature to date surrounding traumatic topics in research suggests that the sensitive nature of the research and what this means for the participants lived experience are the factors that result in participant distress or upset (Condomines & Hennequin, 2014; Mallon & Elliott, 2022). For example, Newman and Kaloupek (2004) in their review of studies found that few participants reported “unanticipated distress” linked to research participation across a wide range of sensitive topics investigated, which included sexual trauma history, mental health, physical injury resulting in hospitalization, and experiences of veterans and refugees. Additionally, Decker et al. (2011) raised the question as to *what* participants actually find distressing, as studies were participants reported upset, where studies that required participants to speak about their trauma for prolonged periods of time (i.e., individual interviews). Thus, posing the question is it how questions are asked, or the length of time participants talk about a distressing topic that primarily causes participant distress or upset.

Indeed, exploration of participation in survey-based research with a “sensitive topic” focus often yields a positive participant response. For example, Cromer et al. (2006) utilized two undergraduate samples in the United States (*n* = 240 and *n* = 277) to compare participants reactions to trauma questions (e.g., sexual and emotional abuse) with their reactions to other potentially invasive questions (e.g., parents’ income, body image, etc.). The findings demonstrated that trauma questions caused relatively minimal distress and were perceived as significantly more important and having greater cost-benefit ratings compared to the other invasive questions. Further, Edwards et al. (2009) reported that out of a sample of 1,056 female U.S. college students, only 43 (4%) reported negative emotional reactions because of their participation in sexual assault research. Their later research (Edwards et al., 2012), which used a sample of 232 male U.S. college students, also demonstrated that only 4.3% experienced immediate negative emotional reactions following their participation, although a 2-month follow-up revealed no lasting impacts.

Moreover, Kirkner et al. (2019) examined the effects of participating in a survey on sexual assault with a diverse sample of 1,863 U.S. adult women who were sexual assault survivors with other trauma histories. The results demonstrate that, overall, participants had a higher positive than negative reaction to survey participation (92%), with a significant proportion indicating that they sought additional support as result of their participation (55%). Further, Yeater et al. (2012) stated that RECs assume that surveys asking questions on “sensitive” topics (e.g., trauma and sex) poses a higher risk to participants than seemingly innocuous measures (e.g., cognitive tests); hence, they tested this assumption by asking 504 U.S. undergraduate students to answer questions on either trauma and sex or on cognitive ability (e.g., tests of vocabulary and abstract reasoning). Participants who completed surveys on trauma and sex, relative to participants who completed the cognitive measures, rated that the study provided higher positive impact and had greater perceived benefits and fewer negative emotional reactions. While participants who completed surveys on trauma and sex reported slightly higher negative emotion reactions than those who completed cognitive measures; however, averages were very low for both groups and outliers were rare in the analysis. Interestingly, all participants rated normal life stressors (e.g., getting a speeding ticket, forgetting Mother’s Day, etc.) as more distressing than participating in the study, which suggests that trauma and sex surveys pose minimal risk. Finally, Yeater et al. (2012) concluded that their study findings suggest that RECs concerns about trauma- and sex-related research, while well intentioned, might be misguided. The above research findings suggest that researching topics such as sexual violence may not be perceived as too “sensitive” despite what some RECs may believe. Indeed, while Decker et al. (2011) raised concerns about the elongated exposure to sensitive topics as a probable negative trigger, Campbell et al. (2010) showcased the potential therapeutic benefits of research when designed with participants at the center. Using a trauma-informed approach, the researchers conducted interviews with survivors on participating in sexual trauma research. Those interviewed found the process helpful, supportive, insightful, useful, healing, and comforting. Therefore, contrary to the “sensitive topic” position, it could be argued that the theme of sexual violence, including own victimization, is not the issue; rather, the methodology and conduct of the research itself is a potential risk that needs to be mitigated. Within the social sciences, we often refer to official codes of conduct designed to primarily guide ethical decision making in relation to harm reduction (Uhlmann et al., 2015). For example, researchers from psychology and social work disciplines have mainly undertaken sexual trauma research to date and regularly subscribe to a risk–benefit ratio (Mortimer et al., 2021). This includes concepts of “integrity” and “justice” adopted within decision making as guiding principles to determine the ratio. Research integrity, while a rather broad and potentially subjective term (Shaw & Satalkar, 2018), most often addresses the procedural and conduct details of a proposed project and researcher (UK Research and Innovation, 2022), whereas justice in the research context reflects the rights of all persons to access and benefit from the contributions of research, maintaining autonomy of choice to participate and share their voice on issues that affect them (American Psychological Association, 2017). Although there may be a risk of participant upset, particularly to those with victimization experience, the risk of silence, ignorance, and inaction in maintaining sexual violence is also large (Ellsberg & Heise, 2005).

While positionality on the issue can, of course, differ depending on which side of the ethical review you are situated, assuming that all survivors are traumatized and vulnerable may in itself be unethical and arguably represents a form of injustice and misrepresentation of survivors (Burgess-Proctor, 2015; Downes et al., 2014; Mulla & Hlavka, 2011). Indeed, feminist scholars including Murphy (2012) and Cunniff Gilson (2016) reflect on the “vexing nature of vulnerability” and its association with perception of weakness or as a “condition of susceptibility to harm,” which potentially only serves as another form of victim blaming. We often reflect on the implications of victims and survivors taking part in research, but have we ever pondered the same concerns for those who have no experience of sexual violence or abuse, are they too “vulnerable or susceptible to harm” for other reasons? And if so, who decides on the “vulnerable” combinations in which to limit or revise research? Moreover, we would be remiss to not consider that our risk aversion, particularly within sexual violence focused research, may in fact be unconsciously informed because of wider sociopolitical positions on this issue.

Historically, institutions have shied away from the topic of sexual violence—research, intervention implementation, policy development, and so on—fearing that engagement with the topic might indicate the presence of a problem and cause untold reputational damage (Cowan & Munro, 2021; Phipps, 2020; Shannon, 2022). The U.K. Higher Education sector is increasingly marketized and competition to attract the greatest number of students is high (Phipps, 2018). This has led to what Phipps refers to as “institutional airbrushing” whereby institutions attempt to minimize or conceal issues related to sexual violence that might negatively impact the institution’s reputation. Alongside Downs (2017), Phipps (2020) describes how sexual violence is recontextualized in economic terms: “the institutional impact of disclosure is projected and totted up” (Phipps, 2020, p. 230) and ignoring or silencing survivors forms a calculated risk. While this calculation might have contributed to the lack of publicly available research, it may have also (un)consciously impacted the ethical review process (Donovan et al., 2020). Even though reputational risk falls outside the remit of RECs, some researchers report instances where research has been rejected for fears of how results might impact the institution (Donovan et al., 2020; Hedgecoe, 2016; Phipps, 2020; Roberts et al., 2007). Whether this view is unfounded is somewhat hard to assess due to the lack of research on the topic but what might allay the fears of RECs could be hearing from participants themselves.

### Present Study

With a solution to the problem of unwanted sexual experiences (USEs) on university campuses unclear (Rosoff, 2018), there is a pressing need to continue research on USEs, particularly in countries such as the United Kingdom, who are only beginning to systemically address this issue (Reynolds et al., 2025). To date, the general conclusion is that while RECs may hold a common belief that asking participants about traumatic events will have a negative impact, this belief has not been fully supported by existing research (Legerski & Bunnell, 2010; Yeater et al., 2012). While some evidence in this area is available, there has been limited focus on the topic of sexual violence and abuse among university students and therefore reduced premise for such important work.

Within the current study, we investigated university students’ reactions to participating in a quantitative survey that examined prevalence rates of USEs. Based on the preceding literature in this research field, the current study aimed to answer the following research questions: (a) Do participants who reported a USE report fewer benefits to participation than those who did not report a USE?, (b) Do participants who reported a USE report higher rates of negative emotional reactions and perceived drawbacks (e.g., questions were too personal) to research participation compared to those who did not report a USE?, (c) Do reactions to sexual trauma questions differ by gender? The above research questions were chosen as they follow up on previous research (i.e., Edwards et al., 2009, 2013; Shorey et al., 2011) and are questions commonly asked by RECs (Edwards et al., 2013). In addition, previous research has neglected to explore if women and men experience different levels of distress or upset from participating in sexual trauma research and the authors are only aware of few studies (Edwards et al., 2009, 2012, 2017) and DePrince & Freyd (2004) who evaluated gender differences of participation in such research. Previous research has shown that women report higher ratings of distress than men on trauma research and women felt such research was a good idea to conduct compared to men (DePrince & Freyd, 2004). Recent research has shown that women and men, who experienced sexual violence, report similar levels of self-blame and self-shame (Farmer et al., 2025); hence, it is important that gender is considered when evaluating trauma research and ethical practices and was included in the analysis of the current study.

## Method

### Procedure

The data utilized in this study come from a larger dataset investigating university students’ USEs across two Northern Ireland (NI) universities (Anyadike-Danes et al., 2023). Participants were recruited through an invitation sent to their university email address and reminders to participate in the survey were sent four times within both universities. The invitation briefly described the research (including its focus on USE), stated the inclusion criteria (over 18 years old and registered at the university), the length of the survey (30–40 min to complete), information about the prize draw incentive (six £50 Amazon gift cards with winners chosen using a random number generator), and a link to the survey hosted on Qualtrics, an online software platform. Potential participants were also invited to complete the survey via social media platforms (see Supplemental Material for recruitment materials). The study was a quantitative survey hosted on Qualtrics, an online software platform, which allowed participants to answer questions in private and at their own pace. The survey launched in November 2020 and closed in April 2021. After the survey closed, anonymized datasets from both universities were merged for further analysis. Due to the nature of the survey, participants were allowed to skip questions if they wished, which led to a large amount of missing data within the main survey dataset. The dataset utilized within the current study is a subset dataset from a wider study, in which the Reaction to Research Participation Questionnaire (RRPQ) measure was an additional request (i.e., students did complete the main quantitative survey and then agreed to complete this measure). For instance, 1,760 participants began the main survey; however, only 1,412 participants completed the demographic questions. The data were screened, and cases were removed prior to analysis if a participant did not complete the Sexual Experiences Survey (SES) and RRPQ. Thus, 469 participants remained for the current study. The study was independently approved by two university RECs: the Faculty of Engineering and Physical Sciences Ethical Committee at Queen’s University Belfast and Ulster University Research Ethics Committee.

### Participants

Participants were 380 female and 89 male university students based at universities in Northern Ireland, with ages ranging from 18 to 57 years (*M* = 24.16, *SD* = 6.386). The majority of participants were White/European (*n* = 418, 89.1%) followed by other ethnic groups (*n* = 48, 10.2%) and prefer not to say (*n* = 3, 0.6%). Participants identified as heterosexual (*n* = 324, 69.1%), bisexual (*n* = 103, 22%), or gay woman/man (*n* = 21, 4.4%), prefer to self-describe (*n* = 13, 2.8%), and prefer not to say (*n* = 8, 1.7%). Most were either single (*n* = 179, 38.2%) or in a relationship (*n* = 149, 31.8%), followed by living with partner (*n* = 69, 14.7%), married/civil partnership (*n* = 34, 7.2%), dating (*n* = 29, 6.2%), separated/divorced (*n* = 5, 1.1%), prefer not to say (*n* = 3, 0.6%), and widowed (*n* = 1, 0.2%). Participants mainly identified their nationality as Irish (*n* = 159, 33.9%) or Northern Irish (*n* = 139, 29.6%), followed by International Student (*n* = 80, 17.1%), British (*n* = 78, 16.6%), English (*n* = 3, 0.6%), Scottish (*n* = 4, 0.9%), Welsh (*n* = 1, 0.2%), dual nationality (*n* = 3, 0.6%), and prefer not to say (*n* = 2, 0.4%), respectively. Finally, most participants were undergraduate students (*n* = 332, 70.7%), with 24.9% of participants (*n* = 117) being in first year, followed by 29.2% (*n* = 137) of participants who were postgraduate/graduate students.

### Measures

#### Sexual Experiences Survey (Short-Form Victimization)

The SES short form victimization (Koss et al., 2007) is a modified version of the SES that estimates participants’ USEs (non-consensual sexual [non-penetrative]) contact, coercion, attempted rape, and penetrative rape (anal, oral, or vaginal) across one time period. The time period measured for this study was “since the students have been at university.” Participants were asked to indicate using the following response items “0,” “1,” “2,” and “3+,” as to how many times they had experienced an USE since they had been a student at an NI Higher Education Institution (HEI). Within the survey, male participants or those with a penis did not complete any questions regarding vaginal penetration, as a skip logic was included in Qualtrics. There are three scoring approaches that can be utilized for the SES, (a) categorical, (b) continuous, and (c) dichotomous. For example, the categorical scoring approach codes the most severe USEs reported by a participant. Thus, if a person indicates a response of 1 to items 1a (unwanted sexual contact via verbal pressure) and to item 4c (completed anal rape via incapacitation), their categorical score would be rape, whereas their continuous score would be 2 and their dichotomous score would be 1 (“victimization present”).

The dichotomous scoring approach was chosen by the authors given findings from previous research (e.g., Anderson et al., 2018), which demonstrated that the dichotomous scoring approach provided the most accurate prevalence rates compared to the categorical and continuous scoring approach. Thus, if a participant endorsed any SES tactic with a “1” (once) or greater, their responses were coded as having experienced at least one USE (i.e., victimization present). Whereas participants who did not endorse any USEs by selecting a score of “0” for any SES tactic, their responses were coded as having no victimization experiences (i.e., no victimization). Furthermore, dichotomous scores were also calculated by category (e.g., sexual contact, coercion, etc.), and these categories were not mutually exclusive (i.e., a participant could be included in two categories). Hence, percentages may not add up to 100% as participants could belong to more than one category.

#### Reactions to Research Participation Questionnaire Revised

The RRPQ-revised is a questionnaire/self-report measure that asks participants for their opinion about what it was like for them to participate in research on that asked them questions about sexual trauma (Newman et al., 2001). Participants were first asked to rank their top 3 reasons for wanting to participate in the study, and following this, participants then responded to 23 items about their reactions to research participation across 5 subscales. The subscales included: Participation Factor assessing cost-benefits (items 14, 15, 17, and 21), Personal Benefits assessing perceived personal insight (items 1, 4, 7, and 13), Emotional Reactions assessing negative emotional reactions (items 3, 5, 10, and 16), Perceived Drawbacks assessing drawback such as whether questions were too personal (items 2, 6, 18, 19, 20, and 22), and Global Evaluation assessing participants’ faith in confidentiality and the researcher’s respect for the individual (items 8, 9, 11, 12, and 23). Further, items 3, 5, 6, 10, 16, 18, 19, and 20 are reverse coded. Items are based on a 5-point scale: *Strongly disagree* (1), *Disagree* (2), *Neutral* (3), *Agree* (4), and *Strongly Agree* (5), with higher scores indicating more positive evaluations. Hence, higher scores on the Perceived Drawbacks and Emotional Reactions indicates less perceived drawbacks and negative emotions, because of participating. The authors (i.e., Newman, personal communication, July 25, 2007; Newman et al., 2001) of the RRPQ recommended that means for each subscale should be computed. Hence, scores on each of the subscales in the RRPQ ranged from 1 to 5. The measure has good internal reliability and validity (Edwards et al., 2009; Kassam-Adams & Newman, 2002; Newman et al., 2001). In the current sample, the internal consistency (Cronbach’s alpha) for the entire scale was .82 and for the subscales was .65 for Participation, .86 for Emotional Reactions, .75 for Personal Benefits, .75 for Perceived Drawbacks, and .82 for Global Evaluation.

### Example of Potentially Distressing Questions from Measures

As previously stated, the current study was undertaken to investigate how participants reacted to participating in research of a sensitive nature, as RECs may be concerned that this type of research will traumatize participants (Yeater et al., 2012), which was the experience of the authors with their individual REC reviews. Thus, the authors believe that it is important to share the types of questions participants were asked in the survey for context (see Table 1 for examples of questions that participants were asked). Participants were asked several personal and potentially distressing questions in the survey about any USEs that they had since they became a student at their university, and following this, they were asked to provide details about the circumstances (e.g., did they know the person, who did they tell, etc.).

**Table 1.** Example of Distressing Questions Asked in Survey.

Scale	Questions Asked
SES (Koss et al., 2007)	2. “Someone tried to put fingers, objects (such as a bottle or a candle), or their penis into my vagina but stopped before genital contact after: a. Telling lies, threatening to end the relationship, threatening to spread rumors about me, making promises I knew were untrue, or continually verbally pressuring me after I said I didn’t want to.
SES (Koss et al., 2007)	3. Someone put their penis into my vagina, or someone inserted fingers or objects into my vagina without my consent: e. Using force, for example holding me down with their body weight, pinning my arms, or having a weapon.

*Note.* SES = sexual experiences survey.

### Data Analysis

All analysis was conducted using IBM SPSS Statistics Version 26 and is based on completed data collected across the key variables. In this analysis, participants were not restricted to reporting only one category USE; thus, they may have selected more than one USE category. Descriptive analysis was conducted to explore the top 3 rank ordered reasons for research participation and the distribution of USEs among participants. *T*-tests were conducted to assess for any significant differences across victimization status in relation to categories of reactions for participation.

## Results

### Descriptive Statistics

The following percentages show the distribution of USEs among the total sample with 58.8% (*n* = 276) reporting at least one USE since starting at university and 41.2% (*n* = 193) reporting no USEs. A gender breakdown of USEs among the sample demonstrated that 62.6% (*n* = 238) of females and 42.7% (*n* = 38) of males reported a USE, whereas 37.4% (*n* = 142) of females and 57.3% (*n* = 51) reported no USEs.

### Assumption Tests

The data were checked for normality, linearity, and outliers, and there were violations of the assumptions detected within the dataset. For instance, Kolmogorov–Smirnov tests were conducted to test for normality distribution for the subscales of the RRPQ and later broken down by gender. Results indicated that the data were not normally distributed as the Kolmogorov–Smirnov tests were significant (*p* < .001). Mann–Whitney U tests were then conducted to assess for any differences across the RRPQ and SES measures for victimization status (i.e., reported victimization versus no victimization experience reported).

### Rank Reasons for Participation

Participants were asked to rank the top 3 reasons as to why they participated in the survey. The first reason (highest ranked) was “To Help Others,” the second reason was “I was curious,” and the third reason was “For the gift card.” Participants were also provided with an “Other” option, where they could type their reason for participating if it was not provided in the options. A few examples of reasons participants provided were as follows: “to help in research,” “imagined there would not be many males who would participate so wanted to participate,” “enjoy completing surveys,” “to be heard,” “wants some good to come from this research,” “to reflect on own experiences,” “felt the research was important,” and “to share own experiences to help others.” Thus, the examples above clearly highlight the vast reasons participants had for choosing to participate in the current study on a sensitive topic.

### Reaction to Research Participation

Means and standard deviations were calculated for each RRPQ subscale and victimization status (see Table 2). Following this, several Mann–Whitney U tests were conducted to determine whether there was a difference in each RRPQ subscale among those who did report a USE and who did not report a USE. Results for the subscales, Participation Factor, Personal Benefits, Perceived Drawbacks, and Global Evaluation did not indicate statistically significant differences among those who reported victimization and no victimization. However, results for the Emotional Reaction subscale indicated a statistically significant difference (*z* = −3.924, *p* < .001), with the means indicating that higher levels of positive emotional reactions among those who reported no victimization (*M* = 3.68, *SD* = 1.01) compared with those who reported victimization (*M* = 3.31, *SD* = 1.02). The findings for those who reported victimization demonstrate that they were neutral for this subscale, which could mean that they did not experience only negative emotions or that they experienced a mixture of emotions. Thus, a Mann–Whitney U test was conducted to determine whether gender would account for the significant difference found for the Emotional Reaction subscale among those who did reported victimization and those who reported no victimization.

**Table 2.** Mann–Whitney U Results for Victimization Status Across RRPQ Subscales.

RRPQ Subscales	Victimization Status	Mean Rank	*z*	Sig
Participation factor	Victimization report	230.20	−0.932	.35
	No victimization	241.86		
Personal benefits	Victimization report	240.23	−1.006	.35
	No victimization	227.53		
Emotional reactions	Victimization report	214.53	−3.924	<.001*
	No victimization	264.28		
Perceived drawbacks	Victimization report	230.81	−0.805	.42
	No victimization	241.00		
Global evaluation	Victimization report	226.79	−1.629	.10
	No victimization	246.75		

*Note*. RRPQ = Reaction to Research Participation Questionnaire.

* Significant at *p* < .001.

Females who did not report victimization had significantly higher scores on the Emotional Reaction (*M* = 3.69, *SD* = 1.02) than those who did report victimization (*M* = 3.27, *SD* = 1.00). The findings demonstrate that those with no victimization did not become distressed during the study; however, those with victimization indicated being neutral, meaning that they did not experience only negative emotions or that they experienced a mixture of emotions.

## Discussion

The current study examined NI university students’ reactions to answering questions about sexual traumatic experiences. There were no differences in reported benefits between those who did and those who did not report a USE. In fact, regardless of victimization status, all participants reported personal benefits from their participation (e.g., gained insight into their experiences); they were glad to have taken part in the current study and felt that the research was for a good cause. These results bridge a gap in the U.K. literature and are consistent with previous research in other countries (DePrince & Chu, 2008; Edwards et al., 2012; Shorey et al., 2011).

In addition, the findings answer RQ2, as participants did not differ on perceived drawbacks, meaning that regardless of whether a participant had or had no experience, neither group found the questions too personal. This finding of no difference between either group is interesting; as shown in Table 1, the current study did ask participants several distressing and personal questions. Participants may not have found the questions distressing because recruitment advertisements and information sheets clearly indicated that they would be asked about sexual trauma (see Supplemental Materials for recruitment advertisements). By informing participants to expect these questions (Fontes, 2004), we may have minimized any distress for participants and, also, ensured that we adhered to the ethical practices within our discipline. Conversely, we must also consider the possibility that participants could have had a delayed reaction to their participation. This was a key focus for Lawyer et al. (2021) who explored immediate and delayed reactions to trauma-related research among sexual violence survivors under controlled conditions. The researchers found initial strong emotional reactions which diminished over time; however, mental health measures included those for post-traumatic stress, anxiety, and depression. While extremely relevant in the context of the topic and lived experience outcome for survivors, within the context of ethics and research participation, it might also be useful to explore more general elements of distress and social functioning linked to research participation that are not holding potential upset (and indeed potential benefits), to clinical levels. Such a longitudinal study may reveal if participants found questions “distressing” in the long term and if reported benefits lasted months following the survey. Such findings could be used to inform best practice for conducting anonymous online surveys on sexual trauma.

Further, results for negative emotional reactions (e.g., became distressed) were only significant for female participants, which answers RQ2 and contradicts previous research (Edwards et al., 2009, 2012; Shorey et al., 2011). The findings demonstrate that female participants, with no victimization experience reported that they had a positive reaction from their participation, whereas female participants who reported a victimization experience reported that they were neutral, meaning that they did not experience *only* negative emotions or that they experienced a mixture of emotions. This finding was not surprising considering the nature of the study coupled with the fact that female students are more likely to participate in sexual trauma research than male and gender non-conforming students (Anderson et al., 2018; Mellins et al., 2017). Further, females are more likely to have disclosed to a friend about their experiences compared to other gender identities (Mennicke et al., 2022), thus potentially finding participating less distressing. The current study sample was mainly female; therefore, without a large sample of males and gender non-conforming students in the current study, these differences could not be examined, which means that their experiences were not well represented. As highlighted in the limitation section below, caution is warranted about the generalizability of the results for other populations given the homogeneity of the sample. Future research should address this gap by ensuring that gender, ethnicity, and sexual orientation minority students are recruited in research on reactions to participation, as it is important to hear their experiences. In addition, the inclusion of open-ended text boxes in the survey or components of qualitative data may have assisted the authors in enriching or contextualizing the findings, particularly around why participants chose neutral on questions relating to their emotional reactions to the study. Thus, the current study can be built upon by future research to address this finding.

The authors hope that the present results will help to further reassure RECs that they do not need to be hesitant in approving sexual trauma research (Clark & Walker, 2011; Edwards et al., 2017), as it offers benefits rather than causing distress among university students (Jaffe et al., 2015). The findings are vital for researchers, participants, and RECs for the following reasons: (a) it builds upon the knowledge base, especially from a U.K. and Irish context, that this type of research does not cause undue distress for participants, and (b) it is beneficial for participants, as they have the opportunity to have their voices heard and share their experiences, with an aim to contribute to an issue that directly impacts them. In fact, participants in the current study were told to expect questions on sexual trauma, and despite knowing this, the findings highlight a desire from participants to help others. Further, completion of the RRPQ was optional, meaning that students could have entered the prize draw without completing it, but many decided to complete it nonetheless.

Thus, considering the results from the current study, if a study utilizes a robust methodology and implements ethical principles within sexual trauma research, RECs should assess these studies fairly. As previously highlighted, the risk of silence, ignorance, and inaction toward sexual violence is also large (Ellsberg & Heise, 2005). RECs should not assume that survivors are traumatized and vulnerable, as researchers have argued that this is a misrepresentation of survivors (Burgess-Proctor, 2015; Downes et al., 2014; Mulla & Hlavka, 2011). Therefore, there is no ethical justification to place addition burdens on or hold sexual trauma researchers to a different standard compared to other researchers (Newman & Kaloupek, 2004; Newman et al., 2001). Hence, the question remains when will RECs tackle these misconceptions on sexual trauma research and cease placing undue burden onto sexual trauma researchers without ethical justification?

### Limitations

Despite the current study’s contribution to the literature on participants’ reactions to sexual trauma research, limitations of this study should be noted. First, the sample for this study was relatively homogeneous in terms of gender, ethnicity, and sexual orientation, despite several attempts made to capture the experiences of gender non-conforming students. However, only a small number (*n* < 12) completed the survey and the RRPQ, which meant that we could only include female and male students within the analysis. Research has demonstrated that non-binary, transgender, and ethnic minority students experience USEs at an increased rate compared to their white, heterosexual, and female counterparts (Reynolds et al., 2025). Further, these students are less likely to have access to the justice system and do not feel welcomed or trust support services; thus, it is vital that future research includes these students to represent their experiences and reactions to sexual trauma research. Second, the current study did not include a follow-up period to investigate whether participants’ reactions changed over time or to determine what, if any, were the long-term effects on participants’ reactions and psychological well-being. Previous research has outlined that longitudinal research is further needed, as some research has documented that when distress is reported, it is temporary (Kirkner et al., 2019; Newman & Kaloupek, 2009). However, there is a dearth of longitudinal research on potential delayed reactions to research participation; thus, it would be helpful for studies to implement a follow-up. In addition, the study did not collect open-ended or qualitative data on participants’ reactions to taking part in the study, which could have enriched or further contextualized the findings. Indeed, anonymous online surveys have been shown to minimize social desirability bias; thus, these surveys are ideal for sexual violence, particularly with university students (Axinn et al., 2018; Berzofsky et al., 2019; Rosenthal & Freyd, 2018). That said, RRPQ questions, which were the focus of the current study, may have resulted in salience bias, whereby sampled participants were more likely to participate and complete the additional survey questions because this was a point of interest or relevance to them (Berzofsky et al., 2019). Greater efforts to explore perceptions of research participation using integrated and mixed methods approaches would be useful in future research in this area.

## Conclusion

Investigating reactions to participation in sexual trauma research has wide-reaching implications for researchers, participants, ethics committees, and practitioners. Such research supports the decision-making process of ethical committees regarding the suitability of research methods, as well as ensuring the safety and wellbeing of potential participants, which is why the current study is vital for RECs. The current study findings may allow RECs to be less apprehensive of sexual trauma research, which would be essential as it will, in turn, allow researchers to further establish a knowledge base that is consistent on the nature, frequency, and outcomes of sexual trauma at an individual and societal level (Jaffe et al., 2015). It is imperative that RECs must reflect on the misconception that all survivors are traumatized and uniquely vulnerable, as this is in itself unethical and arguably a misrepresentation of survivors (Burgess-Proctor, 2015; Downes et al., 2014; Mulla & Hlavka, 2011); thus, we must utilize the current findings to change this conversation. As researchers, it is our ethical responsibility to allow survivors the opportunity to be a part of the conversation when discussing sexual trauma research as outcomes from such research (i.e., trauma-informed approach to research and support services) directly impacts survivors (Campbell et al., 2019). Thus, the onus is now on RECs to utilize the present findings in their future decision making about research on sexual trauma. It is imperative that sexual trauma research is not limited, as this will, in turn, stifle evidence-based practice and support for those most in need (Becker-Blease & Freyd, 2006; Jaffe et al., 2015).

## Supplemental Material

sj-docx-1-jiv-10.1177_08862605251319293 – Supplemental material for Rethinking Sexual Trauma Research: University Students Reactions to Participating in a Sexual Trauma Survey Supplemental material, sj-docx-1-jiv-10.1177_08862605251319293 for Rethinking Sexual Trauma Research: University Students Reactions to Participating in a Sexual Trauma Survey by Megan Reynolds, Ngozi Anyadike-Danes, Susan Lagdon, Áine Aventin, William F. Flack, Emily McGlinchey and Chérie Armour in Journal of Interpersonal Violence
