# Supplementary material for: Rethinking Sexual Trauma Research: University Students Reactions to Participating in a Sexual Trauma Survey
Source: J Interpers Violence. 2025 Feb 18;41(5-6):1130–51. doi: 10.1177/08862605251319293 (PMC12858655; doi:10.1177/08862605251319293)

**Appendix 1: Email Invite to Participate in Survey**

“Dear All, My name is Megan Reynolds, and I am a second year PhD student in the School of Psychology. I am currently recruiting participants for my survey, which investigates adverse life experiences including unwanted sexual experiences (USEs) and mental health outcomes among university students at Queen’s University Belfast. We appreciate this can be a really difficult and sensitive topic to discuss, particularly if you had had such an experience, however the answers you provide will help us to develop future recommendations for support for survivors of these experiences.

You **do not** have to have had an unwanted sexual experience to participate.

If you are interested in participating in the survey, please click on the following: [SURVEY LINK]

The study will take 30-50 minutes to complete and as a thank you for your participation you can enter a prize draw to win a £50 Amazon voucher.

If you have any questions regarding the survey, do not hesitate to contact me (mreynolds16@qub.ac.uk) or my supervisor Professor Cherie Armour ([c.armour@qub.ac.uk](mailto:c.armour@qub.ac.uk))

We are aware that discussion of this topic may be upsetting or cause uncomfortable feelings; if this happens, please consider contacting one of the support organizations listed at the bottom of this email. These same contact details will be available throughout the survey.

Thank you for your time,

Megan Reynolds

If you have been affected by any questions in the survey and would like to seek free confidential advice, the following organisations and support services are available to you:

• QUB Student Wellbeing: 028 9097 2893 or studentwellbeing@qub.ac.uk – Provides support to students regardless if a sexual misconduct incident has happened on or off-campus, or if it happened before you came to Queen’s University. You can also make a report of sexual misconduct to the university at <https://reportandsupport.qub.ac.uk/support/student-wellbeingservice>

• GP: If you are affected by the nature of this study, you can also contact your GP for support.

• PSNI: If you wish to report a sexual violence incident directly to the police call the nonemergency number 101 and you will be put in contact with a specially trained police officer. In an emergency situation please dial the police emergency number 999. You can find more information on the PSNI website, such as making a report online - <https://www.psni.police.uk/crime/sexual-violence-and-abuse/>

• The Rowan SARC: +44 800 389 4424 or http://therowan.net/ – Provides a range of services for people who had been raped or sexually assaulted, such as a forensic medical examination, counselling, and screening for sexually transmitted infections.

• Belfast & Lisburn Women’s Aid: 028 9024 9041 or admin@belfastwomensaid.org.uk or https://belfastwomensaid.org.uk/ - Provides confidential support, information and emergency accommodation.

• Common Youth: 028 9032 8866 or hello@commonyouth.com or https://www.commonyouth.com/ - Provides free, confidential sexual health advice from under 25's, such as emergency contraception, counselling, STI testing and treatment.

• Victim Support NI (Central Office): 02890 243133 or https://www.victimsupportni.com/ – Provides a free and confidential service to individuals affected by a crime.

• Nexus NI (Belfast Office): 028 9032 6803 or Belfast@nexusni.org or https://nexusni.org/belfast-office/ - Provides services and support to people affected by sexual violence in any form.

• Lifeline: 0808 808 8000 or https://www.lifelinehelpline.info/ - Provides a crisis response helpline service for people experiencing distress or despair.

• Samaritans: 028 9066 4422 or http://www.samaritans.org/ - Provides emotional support through a helpline to people experiencing emotional distress, struggling to cope or at risk of suicide.”

**Appendix 2: Social Media Advertisement for Survey**


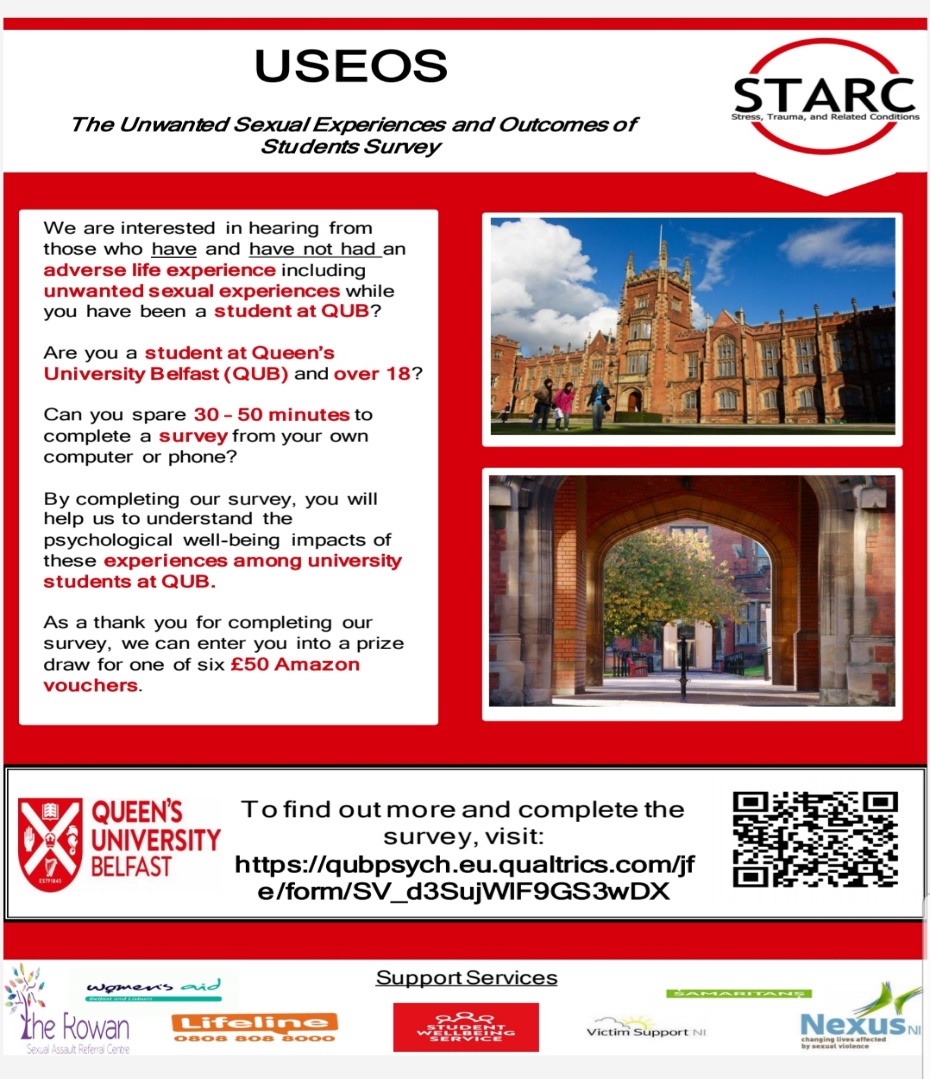

Supplement: sj-docx-1-jiv-10.1177_08862605251319293 – Supplemental material for Rethinking Sexual Trauma Research: University Students Reactions to Participating in a Sexual Trauma Survey [file sj-docx-1-jiv-10.1177_08862605251319293.docx]
